# Formulation of DNA Nanocomposites: Towards Functional Materials for Protein Expression

**DOI:** 10.3390/polym13152395

**Published:** 2021-07-21

**Authors:** Alessa Schipperges, Yong Hu, Svenja Moench, Simone Weigel, Johannes Reith, Diana Ordoñez-Rueda, Kersten S. Rabe, Christof M. Niemeyer

**Affiliations:** 1 Karlsruhe Institute of Technology (KIT), Institute for Biological Interfaces (IBG 1), Hermann-von-Helmholtz-Platz, D-76344 Eggenstein-Leopoldshafen, Germany; alessa.schipperges@kit.edu (A.S.); svenja.moench@kit.edu (S.M.); simone.weigel@kit.edu (S.W.); johannes.reith@partner.kit.edu (J.R.); kersten.rabe@kit.edu (K.S.R.); 2 Department of Polymeric Materials, School of Materials Science and Engineering, Tongji University, 4800 Caoan Road, Shanghai 201804, China; yonghu@tongji.edu.cn; 3 European Molecular Biology Laboratory (EMBL), Flow Cytometry Core Facility, D-69117 Heidelberg, Germany; diana.ordonez@embl.de

**Keywords:** hydrogel, protein expression, nanoparticles, DNA nanotechnology, cellular uptake, cell free protein synthesis, composite materials

## Abstract

DNA hydrogels are an emerging class of materials that hold great promise for numerous biotechnological applications, ranging from tissue engineering to targeted drug delivery and cell-free protein synthesis (CFPS). In addition to the molecular programmability of DNA that can be used to instruct biological systems, the formulation of DNA materials, e.g., as bulk hydrogels or microgels, is also relevant for specific applications. To advance the state of knowledge in this research area, the present work explores the scope of a recently developed class of complex DNA nanocomposites, synthesized by RCA polymerization of DNA-functionalized silica nanoparticles (SiNPs) and carbon nanotubes (CNTs). SiNP/CNT–DNA composites were produced as bulk materials and microgels which contained a plasmid with transcribable genetic information for a fluorescent marker protein. Using confocal microscopy and flow cytometry, we found that the materials are very efficiently taken up by various eukaryotic cell lines, which were able to continue dividing while the ingested material was evenly distributed to the daughter cells. However, no expression of the encoded protein occurred within the cells. While the microgels did not induce production of the marker protein even in a CFPS procedure with eukaryotic cell lysate, the bulk composites proved to be efficient templates for CFPS. This work contributes to the understanding of the molecular interactions between DNA composites and the functional cellular machinery. Implications for the use of such materials for CFPS procedures are discussed.

## 1. Introduction

Starting from the pioneering work of Nadrian Seemann in the early 1980s [1], DNA nanotechnology has developed into a highly innovative and vibrant field of research at the interface of chemistry, materials science, biotechnology and nanotechnology [2,3,4]. The current state of research in DNA nanotechnology ranges from pure “structural DNA nanotechnology” [5] over protein–DNA assemblies [6], nanoparticle-based DNA materials [7,8] and DNA surface technology [9] to DNA-based polymers [10,11,12]. The latter topic was initiated by Dan Luo’s group with the demonstration that branched DNA oligonucleotide building blocks can be assembled into macroscopic hydrogel materials [13]. In addition to production by hybridization/ligation of synthetic oligonucleotides, the DNA polymers can also be produced by enzymatic primer extension, particularly through rolling circle amplification (RCA). This leads to entangled networks of DNA strands that often reveal shape memory persistence [14] and can even be grown enzymatically to mesoscopic structures with distinctive morphologies, often dubbed as “DNA nanoflowers” [15,16]. These nanoflowers are typically 1 to 2 μm in size, and they are readily taken up by mammalian cells, opening up applications for delivery of drugs [16] and proteins [17,18] encapsulated during the polymerization process.

Because DNA can store genetic information, DNA polymer materials can be engineered to instruct biological systems [19,20]. This approach has been used to create protein-producing hydrogels that can direct the enzymatic machinery of cell lysates in vitro to synthesize messenger RNA that is translated into functional proteins [21]. Cell-free protein synthesis (CFPS) is widely considered as a powerful emerging technology that can outperform traditional approaches for the rapid and highly parallel expression of a diverse range of proteins useful for rapid prototyping of biosynthetic pathways and biomanufacturing [22,23]. Therefore, the first demonstration that a DNA hydrogel can be used for the synthesis of 16 different proteins, including membrane and toxic proteins [21], not only attracted much attention but also aroused curiosity as to why such materials are more efficient templates in CFPS than pure plasmids [24].

In subsequent work, special formulations of DNA hydrogels led to microgels generated by polymerization in microfluidic water-in-oil droplets to produce very high local gene concentrations in hydrogels of 1 to 2 μm in diameter for use in CFPS [25]. More recently, formulation as a low-cost hybrid hydrogel prepared with polyethylene glycol diacrylate (PEGDA) and DNA resulted in bulk materials that enable highly efficient and repetitive protein synthesis in CFPS [26]. While it should be noted that CFPS can also occur in artificial hydrogels made from materials other than DNA, such as agarose, clay, hyaluronan, polyacrylamide, fibrin or PEG-peptides [27], the uptake of DNA hydrogels has also been exploited for intracellular production of interfering RNA inside living eukaryotic cells [28].

**Figure 1:**
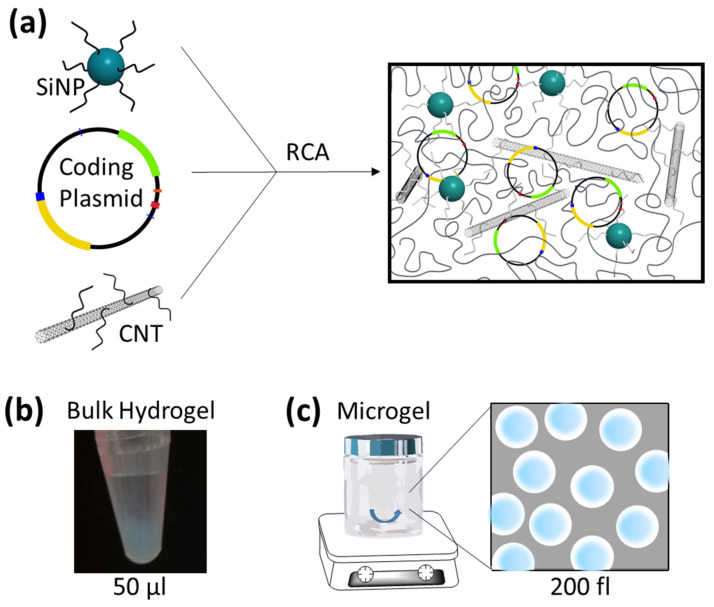
Illustration of RCA synthesis of nanocomposite gels containing an expression vector. (**a**) DNA-modified silica nanoparticles (SiNP) and DNA-modified carbon nanotubes (CNT) are used in different ratios for rolling circle amplification (RCA) interweaving a protein-coding plasmid. Proportions of SiNP (80 nm diameter), CNT (1 µm × 0.83 nm) and coding plasmid (6200 bp) are not sketched to scale. Gels were synthesized in different formats, either in microliter volumes termed as bulk hydrogels (**b**), or inside water-in-oil emulsion droplets (**c**) to yield microgels.

The above-summarized work on bulk- and micro-formulated DNA hydrogels for delivery and synthesis of proteins suggests a significant potential of these materials for application in biotechnology. Therefore, to advance the state of knowledge in this research field, we aimed to investigate the options offered by a new class of complex DNA nanocomposites accessible by RCA polymerization of DNA-functionalized silica nanoparticles (SiNP) and carbon nanotubes (CNT). These SiNP/CNT–DNA composites were recently developed in our group as programmable and cell-instructive biocoatings whose components and mechanical properties can be tailored by varying the concentrations of SiNP and CNT to influence the behavior of living cells [29,30,31]. In addition, these composites are readily taken up by eukaryotic cells and can thus be used for traceable and targeted drug delivery [32]. We report here for the first time the formulation of SiNP/CNT–DNA composites as bulk- and micro-formulated gels containing transcribable genetic information (Figure 1). We show that the gels are very efficiently taken up by various eukaryotic cell lines, but no expression of the encoded protein occurs within the cells. Even in a CFPS procedure with eukaryotic cell lysate, the microgels did not induce protein production. However, the bulk composites proved to be efficient templates for the production of the marker protein, confirming, for the first time, that RCA-based DNA materials can be exploited for this application.

## 2. Materials and Methods

### 2.1. Synthesis of Fluorescent Multifunctional Silica Nanoparticles

Synthesis of multifunctional silica nanoparticles decorated with amino, thiol and phosphonate groups, containing a fluorescent core and with a typical size of 80 nm, was performed according to previous work [33,34]. For synthesis of fluorescent-core particles, the dye STAR 635 NHS-ester (Abberior, Göttingen, Germany) was dissolved in 20% anhydrous dimethylsulfoxide (Merck, Darmstadt, Germany)/80% ethanol (VWR Chemicals, Radnor, PA, USA) (*v*/*v*) at a concentration of 5 mg/mL. Then (3-Aminopropyl) triethoxysilane (APTES, Sigma Aldrich St. Louis, MO, USA) was added at a molar ratio of 10:1 to a final volume of 80 µL. The mixture was incubated under shaking for 16 h. For the synthesis of SiNP, cyclohexane (38 mL, Supelco Merck, Darmstadt, Germany), 1-hexanol (9 mL, Alfa Aesar, Haverhill, MA, USA) and triton X-100 (9 mL, PanReac AppliChem, Glenview, IL, USA) were mixed at 400 rpm in a 250 mL round-bottom Teflon flask. The addition of double-distilled water (2 mL) led to the formation of stable reverse micelles. After stirring for 10 min, the APTES-modified dye (80 µL), tetraethyl orthosilicate (TEOS, Sigma Aldrich, St. Louis, MO, USA) (500 µL) and 30% ammonia solution (500 µL, Supelco Merck, Darmstadt, Germany) were added to the mixture. All following steps were performed protected from light. After 24 h, additional TEOS (250 µL) was added and further stirred for 30 min. Subsequently, 3-(trihydroxysilyl)propyl methylphosphonate (THPMP, Sigma Aldrich, St. Louis, MO, USA) (200 µL) and N1-(3-trimethoxysilylpropyl)diethylenetriamine (DETAPTMS, Sigma Aldrich, St. Louis, MO, USA) (50 µL) were added for modification of the particle surface with negatively charged phosphonate and amino groups. After 24 h of reaction time, (3-Mercaptopropyl)trimethoxysilane (MPTMS, Sigma Aldrich, St. Louis, MO, USA) (30 µL) was added for thiol modification. The mixture was incubated for 3 h before breaking the micelles by adding twice the volume of the mixture in acetone (Merck, Darmstadt, Germany). The precipitated nanoparticles were centrifuged at 17,000× *g*, for 30 min, and washed four times in ethanol absolute and once in double-distilled water. The entire synthesis process for silica nanoparticles was performed at room temperature.

### 2.2. PEGylation and DNA-Modification of SiNP

To modify the surface of the SiNP with PEG-groups, the particles were dispersed in 25% double-distilled water/75% PBS buffer (23 mM KH_2_PO_4_, 77 mM K_2_HPO_4_, 50 mM NaCl, pH 7.4) at a concentration of 10 mg/mL. Poly(ethylene glycol) methyl ether maleimide (mPEG-mal, Sigma Aldrich, St. Louis, MO, USA) (50 mg/mL in DMSO, 10 µL per mL SiNP-1) was added to the nanoparticles. After a one-hour reaction at room temperature, Tris(2-carboxyethyl)phosphine hydrochloride (TCEP) (0.5 M solution, 8 µL per mL SiNP, Alfa Aesar, Haverhill, MA, USA) was added, and the mixture was incubated for 63 h. SiNPs were then washed by centrifugation and re-dispersion in double-distilled water three times. After the last washing step, particles were re-dispersed at 10 mg/mL in 25% ddH_2_O/75% PBS.

For DNA-modification, Glutardialdehyde (50% in water, 250 µL per mL SiNP-2, Merck, Darmstadt, Germany) was added to 10 mg/mL SiNP-2 and stirred for one hour at room temperature. Subsequently, the nanoparticles were washed five times in 25% ddH_2_O/75% PBS and adjusted again to 10 mg/mL. Then ssDNA (aP1, 100 µM, 50 µL per mL SiNP, Sigma Aldrich, St. Louis, MO, USA) was added to the nanoparticles, and the mixture was stirred for 36 h at room temperature. To block unreacted aldehyde groups, glycine (0.4 M, 1 mL per mL SiNP-2, Merck, Darmstadt, Germany) was added as well as sodium cyanoborohydride (60 mM, 400 µL per mL SiNP, Sigma Aldrich, St. Louis, MO, USA) to reduce Schiff’ bases into secondary amines. After one hour of incubation at room temperature, nanoparticles were washed three times by centrifugation and re-dispersion in ddH_2_O with a concentration of 15 mg/mL in the last step. For preservation, 1% penicillin/streptomycin (Gibco, Dublin, Ireland) was added. The entire synthesis process for SiNP–PEG and SiNP–DNA was carried out at room temperature.

### 2.3. DNA-Assisted Solubilization of CNT

First, 1.2 mg single-walled carbon nanotubes (CNT, 0.83 nm average diameter, Sigma-Aldrich, St. Louis, MO, USA) were dispersed in 856 µL ddH_2_O. Then 688 µL of single-stranded DNA (P-2, 100 µM) and 856 µL of NaCl (2 mM) were added. The mixture was sonicated on ice for 3 h, at a power of approximately 10 W, in an Ultrasonic Cleaner (VWR, Radnor, PA, USA). Herein, the sonication process was used to disperse CNT to enable their subsequent wrapping by DNA molecules via π–π stacking interaction. Subsequently, unmodified CNT were separated from the DNA-modified CNT, dubbed as CNT-P, by centrifugation at 16,000× *g* at 4 °C for 90 min. Excess DNA was removed by ultrafiltration in a Vivaspin 500 with a molecular weight cutoff of 50 kDa, at 7000 g and 4 °C, for 3.5 min. CNT-Ps were washed several times by re-dispersing from the membrane using ddH_2_O and centrifugation until no DNA could be detected in the flow through. The concentration of CNT-P was determined by UV–Vis spectrometry at 644 nm using a calibration curve obtained from known concentrations of sodium dodecyl sulfate (SDS)-dispersed CNTs.

### 2.4. Synthesis of Hydrogel by RCA Polymerization

As base for rolling circle amplification (RCA), a circular template was created by ligating linear ssDNA (T-1) on SiNP-P or CNT-P respectively. For this, 5′phosphorylated T-1 (20 µL, 10 µM) was incubated with SiNP-P and CNT-P (individual concentration dependent of SCxx, concentration, as indicated in Supplementary Materials Table S2). Then 10× T4 DNA ligation buffer (5 µL, NEB, Ipswich, MA, USA) was added, and the volume was adjusted to 50 µL with ddH_2_O. During 2.5 h of incubation at 25 °C, 300 rpm, the linear T-1 was circularized on P-1 on the surface of SiNP-P/CNT-P by hybridization. T4 DNA ligase (400,000 U/mL, 1.66 µL, NEB, Ipswich, MA, USA) was added to ligate the previously circularized T-1. The mixture was incubated for another three hours to form particle–primer–template complexes (SiNP-P-T/CNT-P-T). Subsequently, SiNP-P-T/CNT-P-T were used for RCA polymerization. RCA reaction mixture was prepared from dNTP (10 mM, 10 µL, NEB, Ipswich, MA, USA), BSA (1 mg/mL, 5 µL, NEB, Ipswich, MA, USA), 10× phi29 DNA polymerase buffer (500 mM Tris-HCl, 100 mM MgCl_2_, 100 mM (NH_4_)_2_SO_4_, 40 mM DTT, pH 7.5, 5 μL, NEB, Ipswich, MA, USA) and phi29 DNA polymerase (10,000 U/mL, 5 µL, NEB, Ipswich, MA, USA). Then 50 µL of ligation mixture was added to initiate the RCA and the polymerization was performed at 30 °C for 63 h. Subsequently, gels were purified by careful washing with ddH_2_O. For storage, hydrogels were kept at 4 °C.

### 2.5. Synthesis of Microgels by RCA Polymerization

The RCA reaction mixture and the particle–primer–template complexes were prepared as described above. After initiating the RCA by mixing the RCA reaction mixture and particle–primer–template together, the mixture was transferred into 10 times the reactions volume of a mineral oil–surfactant mixture, consisting of mineral oil (Roth, Karlsruhe, Germany), Span 80 (4.5% *vol*/*vol*, VWR, Radnor, PA, USA), Tween 80 (0.4% *vol*/*vol*, Sigma Aldrich, St. Louis, MO, USA) and Triton X-100 (0.05% *vol*/*vol*, PanReac AppliChem, Glenview, IL, USA). By stirring at 1000 rpm, a water-in-oil emulsion was created. After incubation of the emulsion at 30 °C for 63 h, the polymerized microgel was precipitated by the addition of twice the reaction volume of isopropanol. The samples were vortexed thoroughly and centrifuged in a table centrifuge for 10 s. The supernatant was removed, and the microgel was washed three times in isopropanol. The microgel was re-dispersed in ddH_2_O or medium and used immediately.

### 2.6. Synthesis of Hydro- and Microgels Containing Protein-Encoding DNA

For protein expression on the basis of nanocomposite hydrogel, three different plasmids (see Supplementary Materials Figure S1) were added to the RCA reaction mixture, either as plasmid DNA or PCR product, before polymerization. When circularizing T-1 on SiNP-P or CNT-P respectively, no ddH_2_O was added to the reaction mixture. Instead, 42 µL ligation mixture and 8 µL plasmid (10 µg) were added to the RCA reaction mixture. Polymerization into hydro- or microgels was carried out as described above.

### 2.7. Construction of Protein-Encoding DNA

To insert coding DNA into the single stranded RCA nanomaterial, the pcDNA EXP-mKate and pcDNA EXP-eGFP plasmid (Supplementary Materials Figure S1) were opened by PCR, using the primers FP-mKate/RP-mKate and a short 25-base-pair region complementary to the repetitive region of the single strand was added via the FP-mKate primer (rimer, see Supplementary Materials Table S1). For this, a PCR reaction mixture was prepared of 32.5 µL double-distilled water, 10 µL Q5 buffer, 1 µL dNTP (10 mM, NEB, Ipswich, MA, USA), 2.5 µL primers FP-mKate and RP-mKate (10 µM, Supplementary Materials Table S1), 1 µL template (1 ng, pcDNA DEST-mKate or pcDNA DEST-eGFP, Supplementary Materials Figure S1) and 0.5 mL Q5 polymerase (NEB, Ipswich, MA, USA). A touchdown PCR starting at 65 °C was performed by reducing the annealing temperature 0.5 °C for 10 rounds, followed by 20 amplification rounds at 60 °C. The PCR product was purified by using the DNA Clean & Concentrator (Zymo Research, Irvine, CA, USA).

Cloning of eGFP into pT7CFE by Gibson-Reaction: Touchdown PCR was performed with Q5 Polymerase for both the backbone and the insert. The backbone was generated from pT7CFE-CHis, supplied by the CFPS kit with the FP-CFPS 1 and RP-CFPS1 primer (Supplementary Materials Table S1). The eGFP insert was generated from pcDNA EXP-eGFP (Supplementary Materials Figure S1) with the primer FP-eGFP and RP-eGFP (Supplementary Materials Table S1). Backbone and Insert were fused by a Gibson-Reaction by adding a total of 5 µL in equimolar amounts of backbone and insert to 15 µL Gibson-Mix (5× reaction buffer (Tris/HCl, 0.5 M, pH 7,5, MgCl_2_, 50 mM, dGTP, 1 mM, dATP, 1 mM, dTTP, 1 mM, dCTP, 1 mM, DTT, 50 mM, PEG 8000, 0.35 g/mL, NAD, 5 mM, ddH_2_O), T5 Exonuclease, 1 U/µL, Phusion DNA Polymerase, 2 U/µL, Taq DNA Ligase, 40,000 U/µL, ddH_2_O) and incubating for 1 h at 55 °C. The original PCR template was digested by adding 1× cutsmart buffer and Dpn1 for 1 h at 37 °C. Then 2 µL of the reaction mixture was added to 50 µL *E. coli* Dh5α. The bacteria were incubated on ice for 30 min. Heat shock was performed by heating the bacteria to 42 °C in a water bath for 45 s, followed by rapid cooling on ice for 2 min. Then 200 µL of SOC-medium (20 g/l Tryptone, 5 g/L yeast extract, 10 mM NaCl, 2.5 mM KCl, 10 mM MgCl_2_, 10 mM MgSO_4_ and 20 mM Glucose) was added, and the mixture was incubated at 37 °C for 1 h, 600 rpm. Cells were plated on a LB-Agar plate containing Ampicillin and incubated overnight at 37 °C. Several colonies were picked the next day and transferred to 7 mL LB-medium (5 g/L yeast extract, 10 g/L Tryptone and 10 g/L NaCl). After 8 h, a Midiprep (Qiagen, Hilden, Germany) was performed, and the purified plasmid was sent for sequencing to LGC Genomics.

### 2.8. Cell Culture

HeLa cervical cancer cells (ATCC, Manassas, VA, USA) were cultivated in RPMI medium (Gibco, Dublin, Ireland) with 10% FCS (Sigma), 1% GlutaMAX (Gibco, Dublin, Ireland) and 1% penicillin/streptomycin (Gibco, Dublin, Ireland) in 75 cm^2^ tissue culture flasks at 37 °C, 5% CO_2_. Cells were passaged every two to three days. For passaging, cells were washed with DPBS (-/-) (Gibco, Dublin, Ireland) and detached by adding 1 mL 0.05% Trypsine solution (in PBS, 0.02% EDTA, Pan Biotech, Aidenbach, Germany) for 5 min. Trypsin was inactivated by adding 9 mL fresh medium. The concentration of the cells was determined by hemocytometer analysis. For further cultivation, cells were seeded at a concentration of 5 × 104 cells/mL in a fresh 75 cm^2^ tissue culture flask. Human MCF7eGFP breast cancer cells, provided as a gift from the Max-Planck Institute for Molecular Physiology (Dortmund) and A431 cells were incubated in EMEM, with addition of 1% penicillin/streptomycin, 10% FBS, containing an additional 0.6% G418 disulfate salt solution (Sigma, St. Louis, MO, USA). Human MCF7eGFP cells are stably transfected, expressing the EGF receptor fused to an enhanced green fluorescent protein (eGFP). Rat fibroblast cells, REF52 and human embryonic kidney cells, HEK293, were cultured in DMEM with1% penicillin/streptomycin, 10% FBS and 2 mM L-glutamine. Passaging of all cell lines was performed as described for HeLa cells.

### 2.9. Cellular Uptake of Bulk Hydrogels and Microgels

Since the nanocomposites have previously been proven to be highly cytocompatible [29,33], we then used these Materials for cellular uptake studies. Cells were seeded at a density of 8 × 10^4^ cells per dish in a µ-Dish 35 mm (ibidi, Gräfelfing, Germany). After three hours of incubation at 37 °C, 5% CO_2_, 5 µL of a 50% hydro- or microgel/50% cell culture medium solution were added to the supernatant of the cells. After 18 h of incubation, the cells were washed in PBS (-/-) and analyzed by microscopy.

### 2.10. Transfection with Lipofectamine 2000

HeLa cells were seeded at a density of 2 × 10^5^ cells/mL in a six-well plate. Twenty-four hours later, 1.25 µg DNA were incubated in 500 µL OptiMEM (Gibco, Dublin, Ireland) and 2.5 µL Lipofectamine 2000 (Invitrogen, Waltham, MA, USA) for 25 min. Then 400 µL of the transfection mixture was added to the cell.

### 2.11. Cell Staining

Cells were fixated with 4% paraformaldehyde (polysciences, Warrington, PA, USA) for 30 min. The cells were washed three times in PBS, followed by permeabilization with 0.1% Triton X-100 for 5 min. After washing three times with PBS, background fluorescence was reduced by blocking with CAS-Block (Life Technologies, Carlsbad, CA, USA) for 20 min at room temperature. Cells were washed three times in PBS again before staining endosomes by incubating cells in Rab7a antibody (Novus Biologicals, Littleton, CO, USA), 1:200 in 1% BSA in PBS for 1 h at 37 °C. The primary antibody was washed off three times with PBS and cells were incubated in anti-rabbit IgG Atto-488 (Thermo Fisher, Waltham, MA, USA) 1:300 in 1% BSA in PBS for 1 h at room temperature. Alternatively, Aktin was stained by Alexa Fluor 488 Phalloidin (Thermo Fisher, Waltham, MA, USA) 1:40 in PBS for 20 min at room temperature. The cell nucleus was stained with Hoechst (Merck, Darmstadt, Germany), 1:1000 in PBS for 2 min.

### 2.12. Fluorescence Microscopy

Fluorescent microscopy was conducted on an LSM 880 (Carl Zeiss, Oberkochen, Germany), using Zen Black for microscopic recording and Zen Blue for picture analysis.

### 2.13. Flow Cytometry Analysis of Microgel Uptake and Expression of mKate Fluorescent Protein

Cells were incubated with microgels containing pcDNA EXP-mKate for 48 h and then detached from the cell culture flask by adding 200 µL 0.05% Trypsin solution (in PBS, 0.02% EDTA) for 5 min. The trypsin was inactivated by adding 1.8 mL fresh medium. Cells were centrifuged at 1400 rpm for 3 min, and the cell pellet was resuspend in 250 µL of FACS buffer (1× PBS, 2 mM EDTA, 2% BSA). Cell suspensions were filtered through 100 µmm nylon mesh and analyzed by using a BD LSRFortessa™ Flow Cytometer (BD Biosciences) equipped with 5 lasers. Abberior STAR 635 (AS635) was excited by the 635 nm laser, and its emission signal was collected by using a 670/14 bandpass filter. The fluorescent protein mKate was excited by the 561 nm laser and its emission collected with a 610/20 bandpass filter. Post-acquisition analysis was performed with FlowJo software (BD Biosciences, Franklin Lakes, NJ, USA). Experiments and FACS analysis were performed in technical duplicates; a representative experiment is shown.

Cell-free protein synthesis on the basis of nanocomposite hydrogels: The 1-Step Human Coupled IVT Kit-DNA (Thermo Scientific, Waltham, MA, USA) was used for cell free protein synthesis. All components were added according to manufacturer’s instructions to conduct 25 µL reactions in a 384-well plate. Plasmid was either added in solution or incorporated into nanocomposite hydro- or microgel. The reaction was run at 30 °C, in a microplate reader (Synergy MX, BioTek, Winooski, VT, USA), for 2.5 h. Fluorescent intensity was measured at λ_Ex_ 485 nm/λ_Em_ 515 nm every five minutes.

## 3. Results and Discussion

### 3.1. Synthesis and Characterization of Protein-Encoding DNA Composite Materials

To synthesize protein-encoding SiNP/CNT–DNA composite materials as either bulk- or micro-formulated hydrogels, we adopted the recently described method [29]. In brief, SiNP of 80 nm diameter and CNT (0.83 nm diameter, 1 µm length) were functionalized with a 52-mer or 80-mer oligonucleotide primer, respectively, and the resulting DNA-coated particles were used for cyclization of a 98-mer oligonucleotide (for DNA sequences, see Supplementary Materials Table S1). The cyclized oligonucleotide then served as template for enzymatic extension of the particle-bound primers by rolling circle amplification (RCA, Figure 1a). To incorporate the genetic information of the fluorescent proteins mKate or enhanced green fluorescent protein (eGFP), plasmids were added to the RCA reaction mixture either as full-length PCR products or as circular vectors. In addition to the open reading frame of the proteins, the plasmids contained the regulatory elements to enable transcription and translation by the machinery of eukaryotic cells. Detailed maps of the used plasmids, pcDNA EXP-mKate, pcDNA EXP-eGFP or pT7CFE-eGFP, are shown in Supplementary Materials Figure S1.

Both bulk hydrogels and microgels were synthesized from variable amounts of SiNP and CNT, thus leading to composites with variable material properties in terms of entanglement and mechanical stiffness [29]. When the concentration of SiNP was held constant, incorporation of CNT increases the composite’s mechanical stiffness, and vice versa [29]. In particular, we chose a binary composite containing SiNP only (in the following denoted as S100) in addition to ternary CNT-reinforced SiNP–DNA composites with variable mass ratios of SiNP:CNT, ranging from 50:1 (SC 50) over 25:1 (SC 25) to 12.5:1 (SC 12.5) (Supplementary Materials Table S2).

For the production of bulk hydrogels, RCA polymerization was carried in small volumes of about 50 µL, either in Eppendorf cups or wells of a microtiter plate (Figure 1b). After 48 h of polymerization, the hydrogel was carefully washed with deionized water. Microgels were produced in a water-in-oil emulsion by stirring typically about 40 µL of the polymerization mixture in 400 µL of mineral oil solution for 48 h to allow for polymerization inside the water droplets (Figure 1c). The microgel particles were purified by breaking the micelles through addition of isopropanol to the water-in-oil emulsion, collection of the microgels by centrifugation and subsequent washing with isopropanol

**Figure 2:**
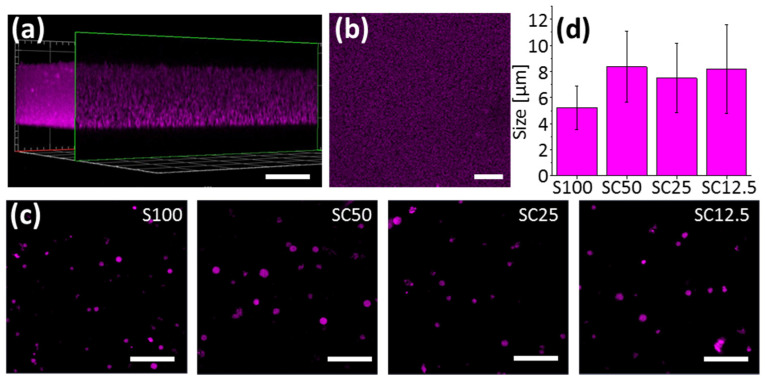
Characterization of bulk hydrogels and microgels. Images in (**a**,**b**) show representative fluorescence micrographs of S100 hydrogel produced by polymerization of 50 µL polymerization mixture in a microplate well: (**a**) 3D reconstruction and (**b**) top view; scale bars are 50 µm. The Images in (**c**) show representative fluorescence micrographs of microgels produced from S100, SC50, SC25 and SC12.5 in a water-in-oil emulsion. Scale bars are 50 µm. The histogram in (**d**) shows the average size of S100, SC50, SC25 and SC12.5 microgels, determined by microscopy. Error bars indicate standard deviations (*n* > 120).

To enable analysis by fluorescence microscopy imaging, both bulk hydrogels and microgels were synthesized as fluorescent nanocomposites by using Abberior STAR 635 (AS635)-labeled SiNP, prepared as previously described [33] to yield the corresponding AS635-labeled composite materials. The prepared materials were then analyzed by both wide-field microscopy and confocal laser scanning microscopy (CLSM). As shown in Figure 2, the fluorescence microscopy images clearly showed the expected morphologies of the materials. The bulk hydrogel prepared in a microplate well formed a homogeneous layer in which the AS635-labeled SiNP were evenly distributed (Figure 2a,b). The microgels appeared as small spherical particles with a diameter of about 5–8 μm (Figure 2c,d). The somewhat larger size of the microgels prepared from the ternary SC materials (≈8 μm), as compared to the binary S100 microgels (≈5 μm), is most likely due to the presence of the stiff CNT, resulting in formation of a less compact material.

### 3.2. Cellular Uptake of DNA Composite Materials

**Figure 3:**
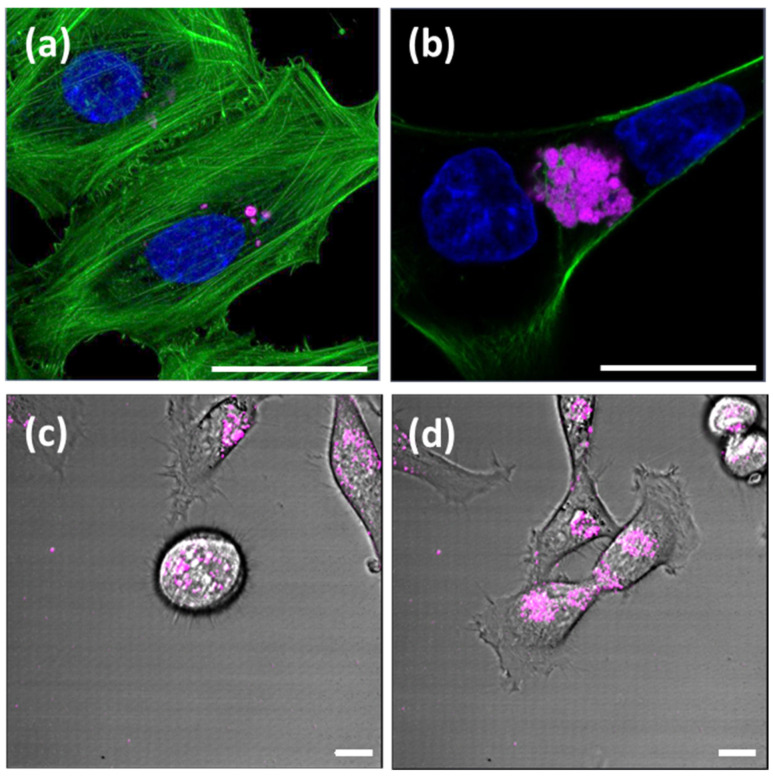
Uptake of S100 composite materials by eukaryotic cells. Cells were incubated overnight in medium containing either bulk- (**a**,**b**) or micro-formulated (**c**,**d**) composite materials. Both bulk hydrogels, S100 (**a**) and SC50 (**b**), as well as S100 microgels (magenta), were taken up by REF52 (**a**), A431 (**b**) and HeLa (**c**,**d**) cells, respectively. REF52 and A431 cells were additionally stained with Phalloidin (green, actin stain) and Hoechst (blue, nuclear stain). All scale bars are 20 µm.

CLSM analysis was then employed to investigate the uptake of DNA composite materials by eukaryotic cells, using HeLa cervical cancer cells, rat fibroblast cells REF52 and human MCF7eGFP breast cancer overexpressing an EGF receptor that is fused to an enhanced green fluorescent protein (eGFP) or the model human epidermoid carcinoma cell line A431. In a typical experiment, about 100.00 cells were seeded in microscopy chambers and 3 h after attachment, 5 µL DNA composite material (i.e., S100 containing 4 mg mL^−1^ SiNP, SC50 containing 4 mg mL^−1^ SiNP and 80 μg mL^−1^ CNT, or SC25 containing 4 mg mL^−1^ SiNP and 160 μg mL^−1^ CNT) were thoroughly mixed with 10 µL cell culture medium and added to the cells. Subsequent to incubation for 24 h, the cells were analyzed by microscopy. We observed that both bulk hydrogels (Figure 3a,b and Supplementary Materials Figure S2) and microgels (Figure 3c,d) were readily ingested by the cells. Z-stack image analyses indicated that the composite materials are located inside the cells (Supplementary Materials Figure S2a,c). Importantly, since the composites are not cytotoxic [29,32], the cells were able to continue dividing, while the ingested material was evenly distributed to the daughter cells (Figure 3c,d; see also Supplementary Materials Video S1).

**Figure 4:**
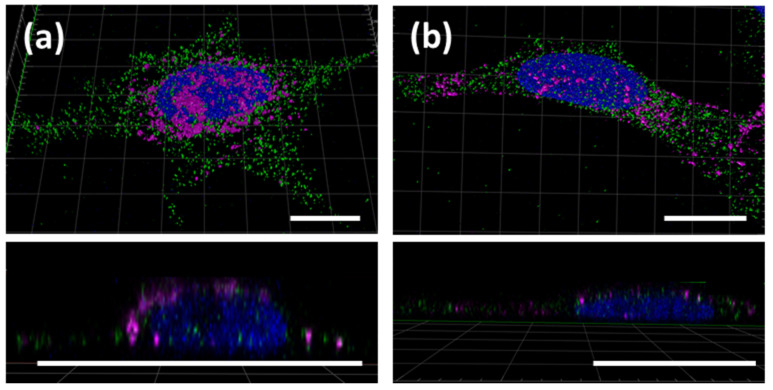
Localization of S100 bulk hydrogel and microgel within HeLa cells. HeLa cells were incubated with S100 hydrogel (**a**) or S100 microgel (**b**) for 24 h (magenta). Endosomes were stained using an anti-Rab7a antibody (**green**), and Hoechst was used for staining of the nucleus (**blue**). All scale bars are 20 µm.

Interestingly, both bulk hydrogels and microgels were observed to migrate to the nucleus and localize there. To further investigate the location of the nanocomposite hydrogels within the cell, bulk hydrogels and microgels of S100 were incubated with HeLa cells for 24 h. Cells were fixed and permeabilized, and endosome staining was performed with an antibody directed against the endosomal marker Rab7a. CLSM analysis did not show any significant co-localization of the Rab7a antibody and either the bulk hydrogel or the microgel (Figure 4a,b). Since no correlation between the localization of the gel and the endosomes within the cells was found, the exact localization and, thus, the uptake mechanism could not be conclusively determined. Previous work on pure DNA hydrogels suggested that uptake could be through receptor-mediated endocytosis or membrane rupture upon unspecific binding [28]. Nonetheless, due to the close proximity of the hydrogels to the nucleus, it seemed possible that components of the nucleus, e.g., polymerases, could migrate into the hydrogel or else that, vice versa, components of the hydrogel leak into the nucleus in order to initiate transcription processes. This hypothesis was to be evaluated in more detail in the following.

### 3.3. Protein-Encoding Hydrogel Materials

**Figure 5:**
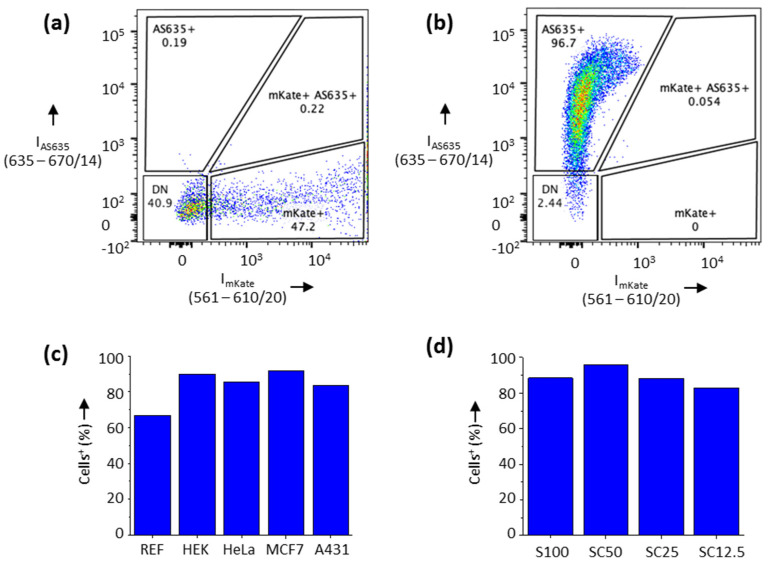
Flow cytometry analysis of the cellular uptake of composite materials. Cells were incubated for 48 h in cell culture medium containing various microgels that contain the expression vector pcDNA EXP-mKate. (**a**) Control in which HeLa cells were transfected with the vector, using Lipofectamine 2000 but no composite materials. (**b**) HeLa cells incubated with the vector-containing SC50 microgel. The dot plot displays single and live cells previously gated based on SSC-A vs. SSC-W signals and negative for DAPI. The bars in (**c**) show the comparison of S100 microgel uptake by various different cell lines. (**d**) Comparison of the uptake of different composite materials by HeLa cells. Standard deviation in technical triplicates for (**c**,**d**) was less than 0.3% for >10,000 cells counted per run.

In order to analyze whether hydrogels containing protein-encoding DNA sequences are similarly well ingested by cells and lead to protein expression, we prepared microgels that contained a full-length PCR product of the vector pcDNA DEST-mKate equipped with a 25 base pair overlap, complementary to the single-stranded regions initiating the RCA process. To quantitatively investigate both the uptake of these microgels by various adherent cell lines, as well as the possible expression of the mKate protein (Figure 5), a systematic study was performed by using flow cytometry. To this end, the cell lines were allowed to ingest various composite materials for 48 h. The cells were then detached from the cell culture vessel, filtered through a 100 µm Nylon mesh to remove cell clusters and analyzed by FACS.

To verify mKate expression after a standard transfection method, a control was first examined, wherein the various cells were transfected with Lipofectamine 2000, using the same pcDNA DEST-mKate vector but not any composite material components (Figure 5a). As expected, the Lipofectamine-mediated control transfection of the vector led to strong fluorescent signals due to expression of the fluorescent mKate protein in 47.2% of the transfected HeLa cells. In contrast, HeLa cells incubated with vector-containing SC50 microgels revealed strong AS635 signals of the material but none of mKate in 96.7% of the cells (Figure 5b). Similar uptake results were obtained with other cell lines and composite materials (Figure 5c,d, respectively; see Supplementary Materials Figure S3 for details), consistently indicating a very good uptake of the microgels by all cell lines (>80%). However, the REF52 fibroblasts showed a slightly reduced uptake capacity (approximately 66%). This agrees well with previously reported data, showing that nanoparticle uptake is reduced in fibroblast cells as compared to cancer cell lines, such as MCF7 [35] or HeLa cells [36]. With respect to material composition, only minor differences occurred, suggesting that SC50 was slightly better ingested than S100 or the other ternary materials. One may speculate that slight differences in material properties could be responsible for the different uptake behavior, as the materials differ in stiffness and surface properties [29].

However, since mKate expression could not be detected in any of the above flow cytometry studies, we decided to increase the sensitivity of detection by utilizing the vector pcDNA EXP-eGFP, which encodes the enhanced green fluorescent protein (eGFP) that has a brightness more than twice of that of mKate [37]. SC25 microparticles bearing linearized PCR products of this vector were readily ingested; however, no eGFP fluorescence was detectable in fluorescence microscopy analyses (Supplementary Materials Figure S4a,b). The same results were obtained with microgels containing whole plasmid DNA instead of PCR products (Supplementary Materials Figure S4c,d). Hence, the combined results of flow cytometry and CLSM thus showed very clearly that the genetic information for fluorescent proteins contained in the nanocomposite is not transcribed and translated by the machinery of eukaryotic cells. Thus, the close proximity of the composite materials to the nucleus is not sufficient to overcome the physical barrier between the compartments, so that transcription of DNA into mRNA can occur, which usually requires the localization in the nucleus [38].

**Figure 6:**
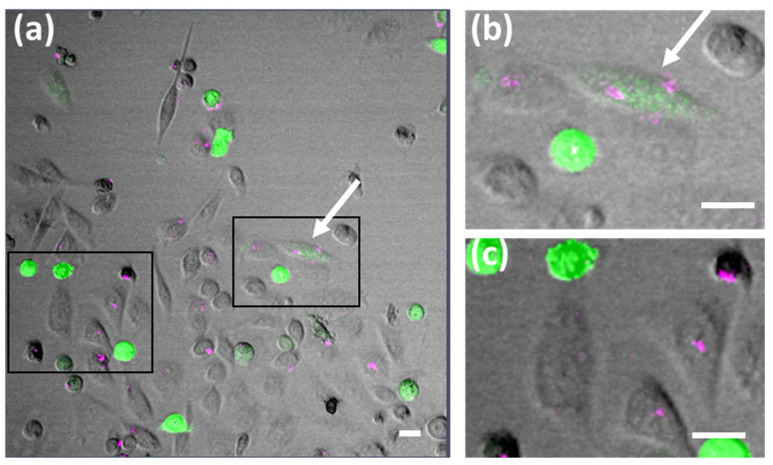
CLSM analysis of eGFP expression in HeLa cells based on nanocomposites preincubated with Lipofectamine 2000. SC25 microgels containing pcDNA DEST-eGFP were treated with Lipofectamine 2000 for 20 min and then added to HeLa cells for 24 h. Magnifications of the boxed areas (**a**) are shown in the insets on the left hand side (**b**,**c**). Only very few cells show both signals (**b**, white arrow). All scale bars are 20 µm.

We also performed uptake experiments by using bulk hydrogels and microgels made of SC25, which were incubated with Lipofectamine transfection reagent prior to their administration to HeLa cells (Figure 6 and Supplementary Materials Figure S5). It was hypothesized that treatment of the microgels with Lipofectamine might lead to formation of particles that could cross the nuclear membrane and thus serve as a template for transcription of mRNA within the nucleus. Indeed, microscopy analysis revealed the presence of GFP-expressing cells within seven hours after transfection. However, only very few fluorescent cells also contained the gel materials. Most cells showed either eGFP or AS635 fluorescent signals. These results indicated, on the one hand, that the vector was not damaged during the polymerization procedure. On the other hand, the absence of simultaneous AS635 and eGFP signals within a cell suggested that only portions of vector not incorporated into the materials can be transcribed by the cells. Free, unincorporated vector may result either from failure to incorporate during polymerization or from subsequent detachment from the material. Thus, no clear indication of cellular transcription/translation of the hydrogel-linked vector could be obtained from these experiments either.

### 3.4. Cell-Free Protein Synthesis with DNA Composite Materials

**Figure 7:**
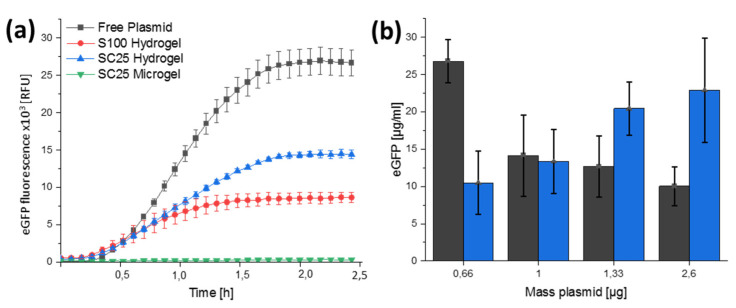
Cell-free protein synthesis on the basis of nanocomposite hydrogels. Cell-free protein synthesis was performed at 30 °C for 2.5 h in 25 µL reactions, using a HeLa-cell-extract-based system following manufacturer’s instructions. (**a**) The expression vector was added incorporated into S100 (**red**) or SC25 (**blue**) hydrogel or SC25 microgel (**green**). As a control, 0.66 µg plasmid was used in solution (**black**), corresponding to the amount of plasmid incorporated into the hydrogel. Fluorescence measurement at λ_Ex_ 485 nm/λ_Em_ 515 nm shows the highest level of eGFP expression for the plasmid control sample. (**b**) Equal amounts of the expression vector were incorporated either into four 2.5 µL SC25 (**blue**) hydrogels or used as free plasmids in solution (**black**). Fluorescence measurement at λ_Ex_ 485 nm/λ_Em_ 515 nm enables calculation of the level of eGFP expression based on an eGFP calibration curve.

To further explore the scope of the protein-encoding DNA composites, both the bulk hydrogels and microgel materials were used in CFPS experiments. To this end, the open reading frame of eGFP was inserted into the pT7CFE vector, which is provided with the commercial HeLa-based CFPS Kit (Supplementary Materials Figure S1). The pT7CFE-eGFP was then incorporated into S100 hydrogel, SC25 hydrogel and SC25 microgel. To conduct CFPS experiments, 5 µL of each material was incubated with HeLa cell extract according to manufacturer’s instruction and for comparison, an equal amount of the pure plasmid was used as control. CPFS of eGFP was followed by recording the emerging fluorescence of eGFP at λEm515 nm, in a microtiter plate (Figure 7A). While we observed eGFP production for both hydrogel variations, no eGFP production was detected for the microgels. In fact, the free plasmid control showed the highest eGFP expression, while this was reduced to 68% in the samples containing protein-encoding SC25 composite material and to about 46% in the case of the S100.

Since the same amounts of plasmid were used in all reactions, the reduced eGFP expression might result from a limited accessibility of the plasmid inside the nanocomposite materials. To elaborate on this hypothesis, we measured the eGFP expression in dependency of increasing amounts of plasmid by using either the free plasmid or plasmid incorporated inside SC25 bulk hydrogel material (Figure 7B). As expected, the eGFP expression dropped when the amounts of free plasmid exceeded the maximum recommended by the manufacturer (about 1 µg in a typical 25 µL reaction). However, in the case of plasmid encapsulated inside the SC25 material, an increase in eGFP expression was still evident. These results suggest that the effective plasmid concentration is reduced due to incorporation into the hydrogel material and/or that the interfering influence of excessive DNA concentrations on CFPS is reduced by the hydrogel material.

With respect to the nature of the composite materials, the results also indicated, on the one hand, that the formulation of the microgels used here is not suitable to yield functional materials for employment in CFPS. This could be due to the fact that washing steps with isopropanol are used in the purification of the microgels, which could cause condensation and/or denaturation of the plasmids. On the other hand, the results obtained for the S100 and SC25 hydrogel materials clearly show that the integration of the plasmids into these composites may very well result in functional materials for CFPS. The marked improvement in CFPS efficacy observed with SC25 as compared to S100 could be due to altered mesh sizes and entanglement characteristics due to the integrated CNTs [29], which could improve the accessibility of genetic material to the components of the transcription/translation machinery.

## 4. Conclusions

To advance the state of knowledge in the research on functional DNA materials for the synthesis of proteins, this work was intended to explore the scope of a recently developed class of complex DNA nanocomposites, accessible through RCA polymerization of DNA-functionalized silica nanoparticles (SiNPs) and carbon nanotubes (CNTs). To this end, SiNP/CNT–DNA composites bearing a plasmid with transcribable genetic information for a fluorescent marker protein were produced as bulk materials and microgels. High uptake efficacies were observed for various eukaryotic cell lines. Importantly, it has been shown for the first time that the ingested material is transported between different cells and also distributed equally among daughter cells during cell division (Figure 3 and Supplementary Materials Video S1). While the ingested material was often localized in close proximity to the nucleus, production of the fluorescent marker protein was not observed to commence. Therefore, it seems unlikely that components of the nucleus, e.g., polymerases, migrate into the hydrogel or, conversely, that components of the hydrogel enter the nucleus to initiate transcription of the mRNA. This result is consistent with previous work on an RNA-producing DNA hydrogel as a platform for a high-performance RNA interference system [28] in which a bacterial RNA polymerase was encapsulated in a DNA hydrogel to facilitate RNA production in eukaryotic cells.

In contrast, the SiNP/CNT–DNA composites formulated as bulk materials proved to be efficient templates in cell-free protein synthesis (CFPS), using a eukaryotic cell lysate. The results obtained gave clear indications that the composition of the materials (S100 vs. SC25, in Figure 7a) influences the efficiency of CFPS. Further studies involving pure DNA hydrogels will show whether and to what extent the presence of SiNP and CNT influence the molecular accessibility of the genetic material for the components of the transcription/translation machinery. The results in Figure 7b indicate that an important control in such studies is the direct concentration-dependent benchmarking with free plasmid. Here, our results suggest that a possible interfering influence of too-high plasmid concentration could be overcompensated by packaging the DNA template into the condensed RCA materials, so that the effective free concentration of transcribable DNA is lower. In addition to underlining the potential of DNA materials for biotechnological applications in CFPS, our work also has relevance to the current developments of DNA-modifying enzymes, such as DNA polymerases and Cas9, for biosensing applications [39] or the ongoing development of carbon and silica-based nanomaterials as emergent platforms for theranostics [40,41].

## Data Availability

Data supporting the findings of this study are available within the paper and its Supplementary Materials files. All other relevant data are available from authors upon reasonable request.

## Supplementary Materials

The following are available online at https://www.mdpi.com/article/10.3390/polym13152395/s1. Figure S1: Plasmid maps for vectors used in FACS experiments (a), microscopy (b) and cell-free protein expression (c). Figure S2: Uptake of nanocomposite hydrogel by HeLa and MCF7 cells. Figure S3: Microgel uptake and mKate expression in various eukaryotic cell lines. Figure S4: CLSM analysis of HeLa cells containing composite materials bearing DNA coding for eGFP. Figure S5: CLSM analysis of eGFP expression in HeLa cells that were treated with SC25 containing pcDNA EXP-eGFP and preincubated with Lipofectamine 2000. Video S1: Ingested microgel materials are distributed into daughter cells upon cell division Table S1: List of DNA Sequences. Table S2: Overview of SiNP-P/CNT-P–DNA nanocomposite materials.
