# Supplementary material for: Formulation of DNA Nanocomposites: Towards Functional Materials for Protein Expression
Source: Polymers (Basel). 2021 Jul 21;13(15):2395. doi: 10.3390/polym13152395 (PMC8347857; doi:10.3390/polym13152395)
Supplement: Supplementary file 1 [file polymers-13-02395-s001.zip › 210629 Composite Formulation_SI.pdf]

Formulation of DNA Nanocomposites: From Microgels to Functional Materials for Protein Expression

Alessa Schipperges, Yong Hu, Svenja Moench, Simone Weigel, Johannes Reith, Diana Ordonez, Kersten S. Rabe, Christof M. Niemeyer*

Supporting Information

Supplementary Figures

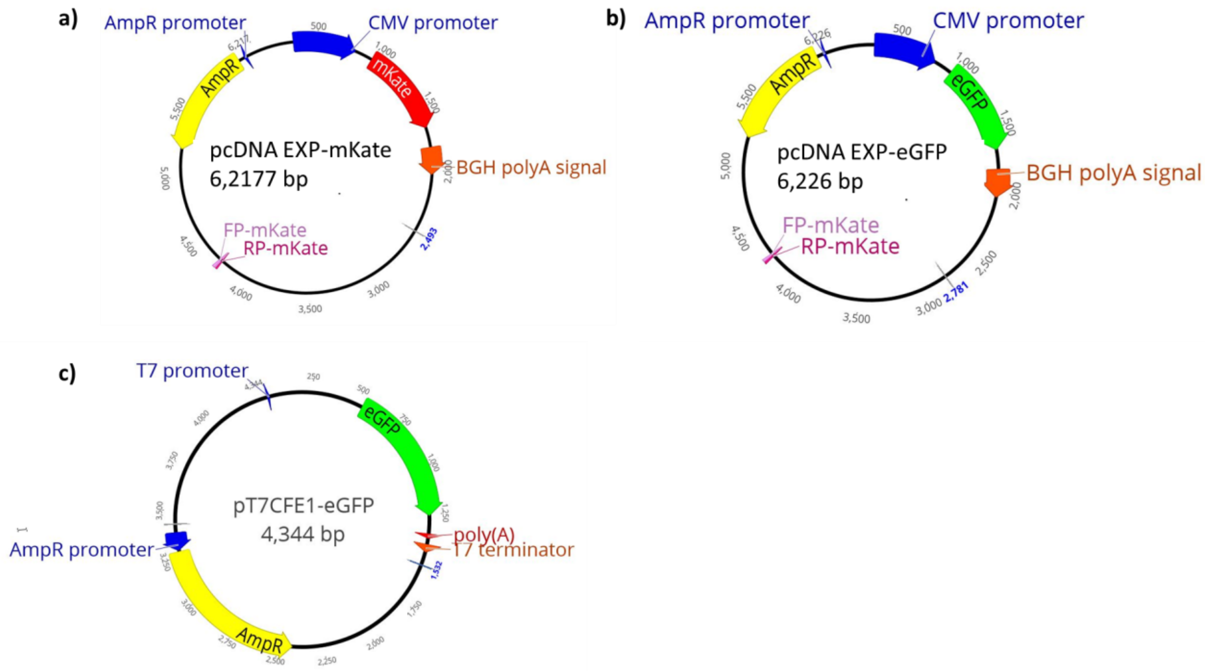

Figure S1: Plasmid maps for vectors used in FACS experiments (a), microscopy (b) and cell free protein expression (c). pcDNA EXP-mKate and pcDNA EXP-eGFP were used to transfect HeLa cells for FACS and microscopy experiments, respectively. The fluorescent protein is under the control of a CMV promoter and terminated by a BGH polyA signal. Vector pT7CFE-eGFP was used for cell free protein expression in a HeLa based system. The fluorescent protein here is under the control of a T7 promoter, followed by a poly(A) signal and terminated by a T7 terminator. All three vectors contain an Ampicillin resistance under the control of an AmpR promoter.

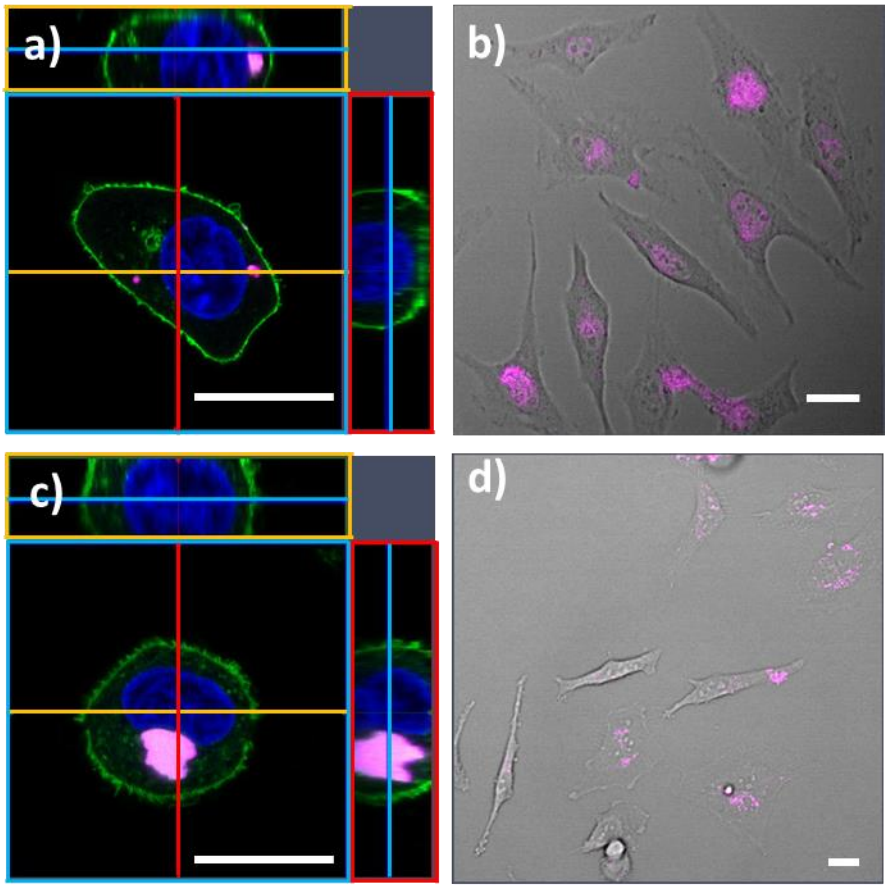

Figure S2: Uptake of nanocomposite hydrogel by HeLa and MCF7 cells. Cells were incubated overnight in medium containing different nanocomposite hydrogels (magenta). HeLa cells with ingested S100 (a) and SC25 (b) and $MCF7_{eGFP}$ cells with ingested S100 (c) and SC50 (d) are shown. The $MCF7_{eGFP}$ cells stably overexpress the EGF receptor fused to eGFP (green), which is localized at the cell membrane. Hoechst staining of the nucleus (blue) allows to determine the relative position of the hydrogel inside the cells by Z-stack image analyses. In c) and d) the main frame (light blue box) is part of a Z-stack, reconstructed to a 3D confocal image. Vertical or horizontal cross sections through the middle of the cell (red and yellow lines, respectively) lead to 3D images shown in the red and yellow boxes on top and right hand side, respectively. Note that the hydrogels are located inside the cell. All scale bars are 20 µm.

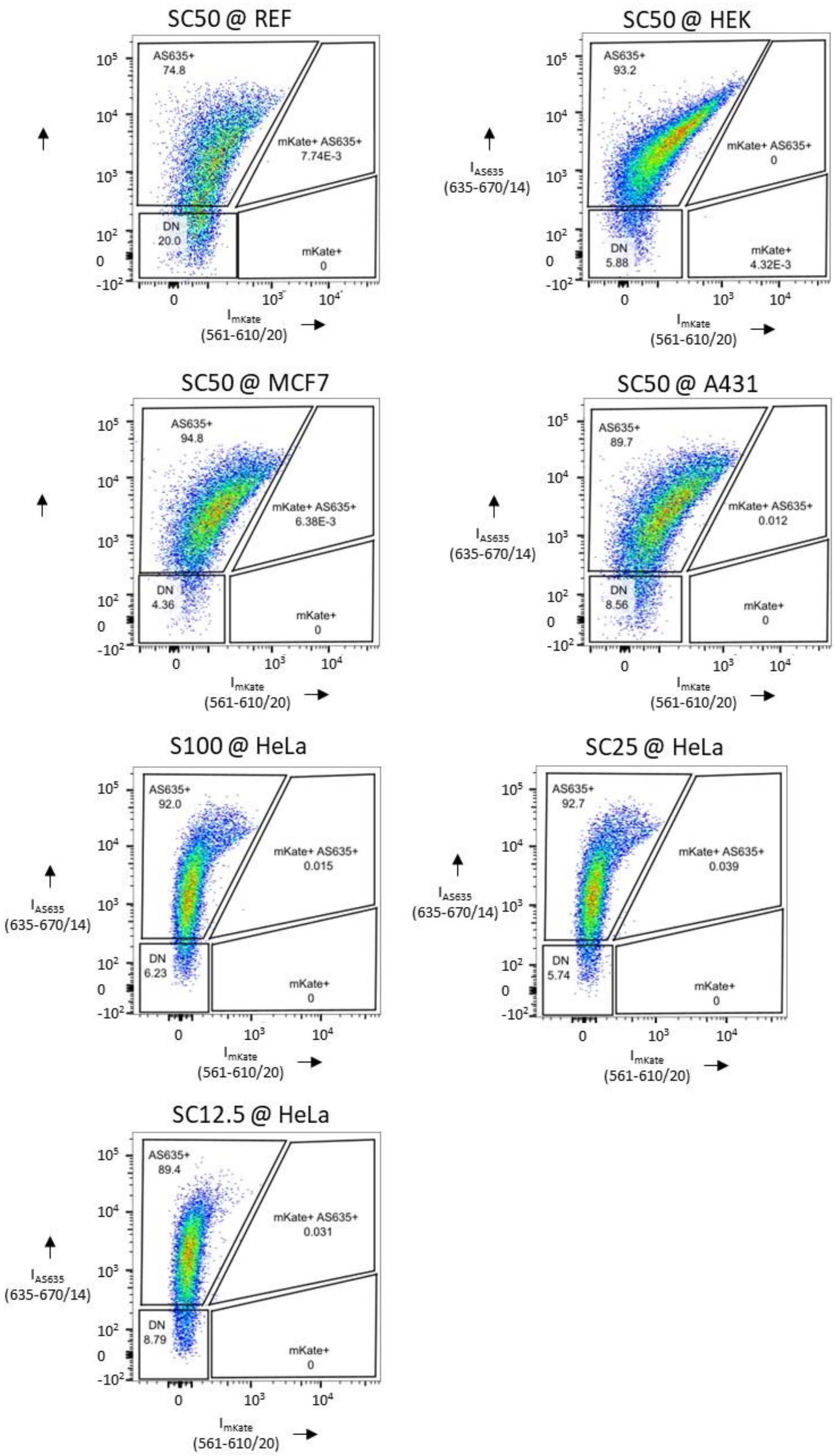

Figure S3: Microgel uptake and mKate expression in various eukaryotic cell lines: Various cell lines (@cell line) were incubated with different kind of microgels (SCxx) for two days. Before FACS analysis, cells were detached from their well, washed and filtered. >10 000 cells were counted per run. Strong AS635 fluorescence confirms the presence of the microgel. Weak mKate signal indicates no substantial expression of the fluorescent protein. The dot plots display single and live cells previously gated based on SSC-A vs SSC-W signals and negative for DAPI.

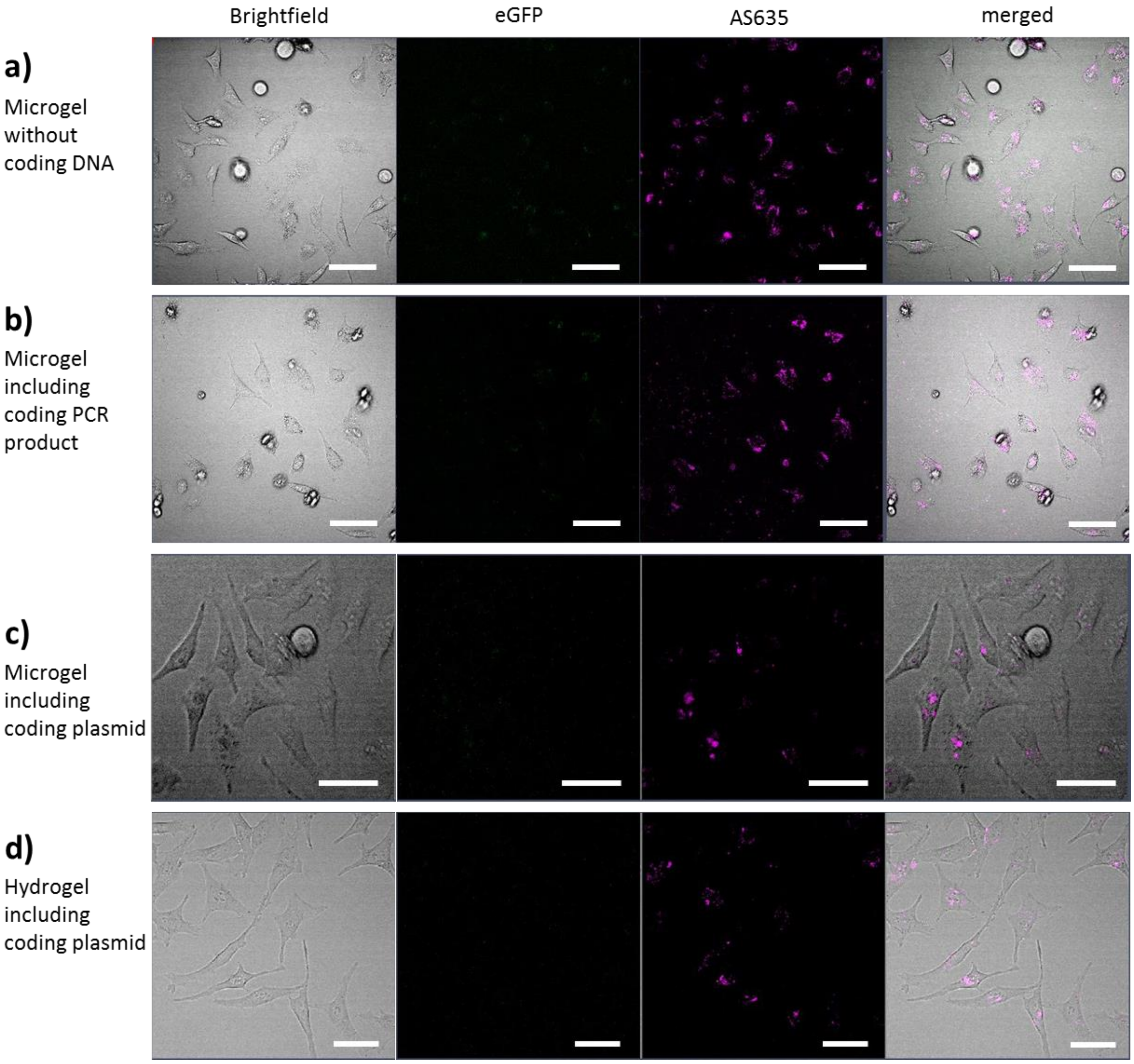

Figure S4: CLSM analysis of HeLa cells containing composite materials bearing DNA coding for eGFP. 5 µl SC25 Microgel (a-c) or Hydrogel (d) containing either no coding DNA (a), a PCR product of the plasmid pcDNA DEST-eGFP (b) or the plasmid pcDNA DEST-eGFP itself, were added to the medium of 100 000 HeLa cells. After 48 hours, cells were washed and analyzed by CLSM. Composite materials (magenta) can be seen inside the cells (merged) but no eGFP expression (green) can be detected in any coding DNA/nanocomposite material combination. Scale bars are 50 µm.

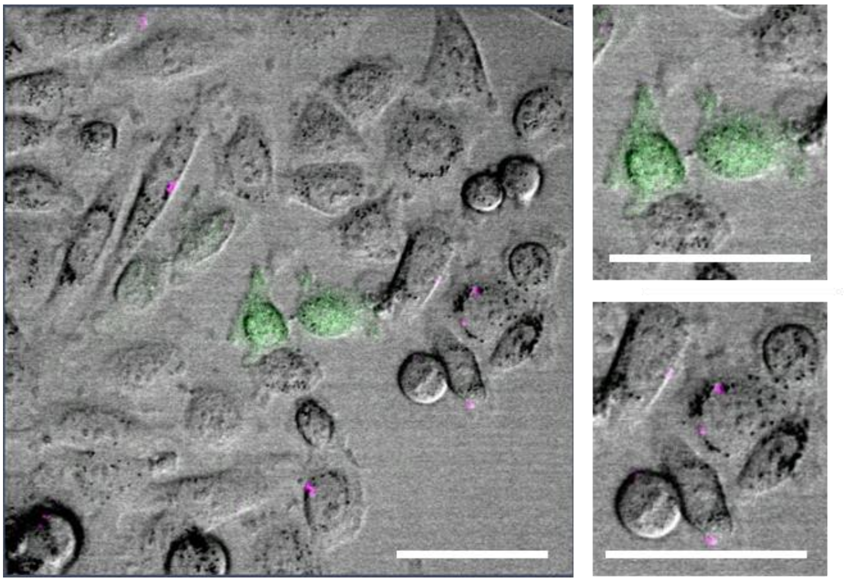

Figure S5: CLSM analysis of eGFP expression in HeLa cells that were treated with SC25 containing pcDNA EXP-eGFP and preincubated with Lipofectamine 2000. 5 µl SC25 Hydrogel with pcDNA EXP-eGFP were incubated with Lipofectamine 2000 for 20 min and added to 100 000 HeLa cells growing in 2 ml medium in ibidi microscopy chambers. After 24 hours, cells were washed in PBS and eGFP expression was determined using CLSM. Microscopic analysis does not show a correlation between eGFP expression (green) and microgel uptake (magenta) with most cells either showing eGFP expression or microgel uptake. Scale bars 50 µm.

Supplementary Videos

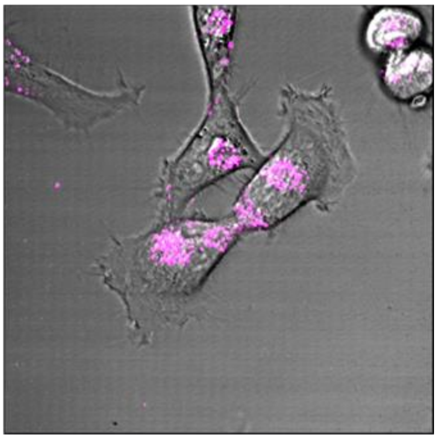

Video S1: Ingested microgel materials are distributed into daughter cells upon cell division. HeLa cells were incubated overnight in medium with S100 microgel (magenta). After 18 hours, a time lapse experiment was started, taking pictures every 5 minutes for five hours. Several cells containing microgel can be seen, two of them undergoing division. The microgel in the dividing cells is equally distributed between the two daughter cells. Sporadically, cells can be observed exchanging microgel with neighboring cells.

Supplementary Tables

Table S1: List of DNA Sequences

Name	Sequence (5´ - 3´)	Modification
aP1	TCTAACTGCTGCGCCGCCGGGAAAATACTGTAC GGTTAGA	5´-Amine C12
P2	TTTTTTTTTTTTTTTTTTTTTTTTTTTTTTTTTTTTTTT TTTCTAACTGCTGCGCCGCCGGGAAAATACTGTA CGGTTAGA	
T	TTCCCGGCGGCGCAGCAGTTAGATGCTGCTGCA GCGATACGCGTATCGCTATGGGTAACCGTACGG TTACCCGCAGCAGCATCTAACCGTACAGTATT	5’ Phosphorylation
FP - CFPS 1	GAGCTGTACAAGTAAATGGGATCCGAATTCGATA TCTTAATTAAGCTGCA	
RP – CFPS 1	CTCGCCCTTGCTCACATGGGTGGTGGCCATATTA TCATCGTGTTT	
FP - eGFP	GTGAGCAAGGGCGAGGAGCTGTTCAC	
RP – eGFP	TTACTTGTACAGCTCGTCCATGCCGAGAGT	
FP-mKate	CGCGGCGGCCCTTTTATGACGCGGTTTGCGTAT TGGGCGCTCTTCC	
RP-mKate	CTCTCCCCGCGCGTTGGCCG	

Table S2: Overview of SiNP-P/CNT-P-DNA nanocomposite materials

**Name** (mass ratio of SiNP-P:CNT-P)	**SiNP-P** [µg/ml]	**CNT-P** [µg/ml]
S100	4000	-
SC50	4000	80
SC25	4000	160
SC12.5	4000	320
